# The Development of a New Setup for Video-assisted Thoracic Surgery

**DOI:** 10.1155/DTE.6.43

**Published:** 1999

**Authors:** Masahiro Tsuboi, Harumasa Sakai, Shinichi Nagata, Takafumi Kono, Kinya Furukawa, Chimori Konaka, Harubumi Kato

**Affiliations:** Department of Thoracic Surgery Tokyo Medical University Hospital 7-1, Nishishinjuku 6-Chome Shinjuku-ku Tokyo 160-0023 Japan

## Abstract

In order to accomplish video-assisted thoracic surgery (VATS) in a much easier and safer way, especially for assistant operators, we have developed a new display system for VATS. The original thoracoscope has been designed for this new system. The monitor is fixed at approximately 10 cm away from the surface of the chest wall just above the operative field. In using this procedure, the operator and assistants can see the patient and the monitor at the same time. According to this new idea, the previous problem in the area of hand–eye coordination and the three-dimensional understanding of this procedure can be improved compared to the image of the conventional thoracoscopy, because it is not necessary for the operator and assistants to look up at the monitors. When the thoracoscopy was placed in an adequate position to resect the target pathology, this new system led to good and easy handling of instruments, as it was with the standard thoracotomy.


*Diagnostic and Therapeutic Endoscopy*, Vol. 6, pp. 43–46
Reprints available directly from the publisher
Photocopying permitted by license only

© 1999 OPA (Overseas Publishers Association) N.V.
Published by license under
the Harwood Academic Publishers imprint,
part of The Gordon and Breach Publishing Group.
Printed in Malaysia.

### The Development of a New Setup for Video-assisted Thoracic Surgery

MASAHIRO TSUBOI*, HARUMASA SAKAI, SHINICHI NAGATA, TAKAFUMI KONO, KINYA FURUKAWA, CHIMORI KONAKA and HARUBUMI KATO

*Department of Thoracic Surgery, Tokyo Medical University Hospital, 7-1, Nishishinjuku 6-Chome, Shinjuku-ku, Tokyo 160-0023, Japan*

*(Received in final form 14 May 1999)*

**In order to accomplish video-assisted thoracic surgery (VATS) in a much easier and safer way, especially for assistant operators, we have developed a new display system for VATS. The original thoracoscope has been designed for this new system. The monitor is fixed at approximately 10 cm away from the surface of the chest wall just above the operative field. In using this procedure, the operator and assistants can see the patient and the monitor at the same time. According to this new idea, the previous problem in the area of hand–eye coordination and the three-dimensional understanding of this procedure can be improved compared to the image of the conventional thoracoscopy, because it is not necessary for the operator and assistants to look up at the monitors. When the thoracoscopy was placed in an adequate position to resect the target pathology, this new system led to good and easy handling of instruments, as it was with the standard thoracotomy.**

*Keywords:* Hand–eye coordination, Setup, Thoracoscope, Video-assisted thoracic surgery (VATS)

### INTRODUCTION

Video-assisted thoracic surgery (VATS) is increasingly replacing the standard thoracotomy for many intrathoracic diseases [1]. This technique has already proved to be feasible [2–5]. However, both the visual field and handling of instruments should be considered technically as limiting factors for this particular procedure, because the field of operative vision under VATS is indirectly applied by the thoracoscopy. In order to accomplish this procedure in a much easier and safer way, especially for assistant operators, we have developed a new display system for VATS. We have introduced here our new display system and described its advantages and some considerations of our system based on the preliminary data.

* Corresponding author. Tel.: 81-3-3342-6111. Fax: 81-3-3349-0326. E-mail: mtsuboi@za2.so-net.ne.jp.

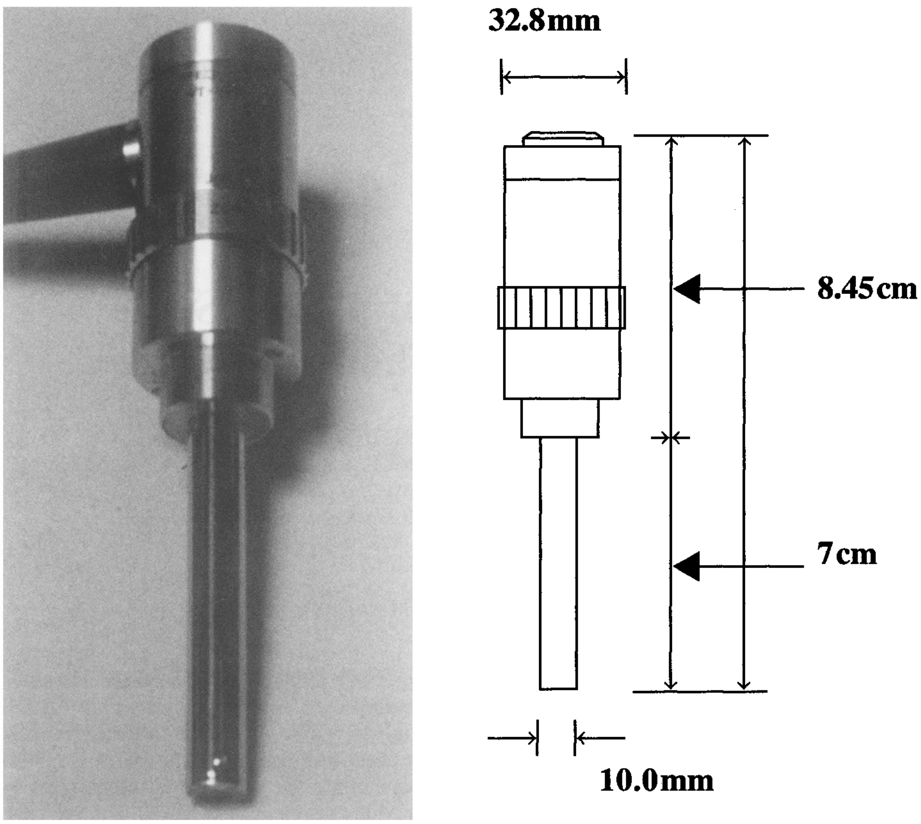

FIGURE 1 The features of the original thoracoscope are as follows: (1) total length is 15.45 cm, (2) the inserted part is 10 mm in diameter and 7 cm in length, (3) the visual angle of the scope is between 60° and 120°, (4) the visible depth is between 25 mm and remains limitless.

### MATERIALS AND METHODS

*Thoracoscope* The original thoracoscope has been designed for this new system (Fig. 1). In practical use, this scope is inserted and set up above the center of the operative field with pathological target. In the thoracic cavity, only the tip of the scope can be seen.

*Monitor* The color-flat display (Color Flat Pannel; Matsushita Electric Industrial Co., Ltd. Japan) has been used as the monitor.

*Setup* The monitor is fixed at approximately 10 cm away from the surface of the chest wall just above the operative field (Fig. 2). By using the zoom function of the new scope, both the size and the direction of this image resemble those of the structures which can be seen under conventional methods.

*Investigation plan* In order to evaluate the usefulness of this new display system, we performed the experimental VATS studies in three pigs and compared the conventional system to the new one. In every experiment, partial resection and right upper lobectomy were performed by three testers. Each tester classified his judgment into four aspects.

### RESULTS

Although no one estimated this new system to be an excellent one, about 60% of nine testers evaluated

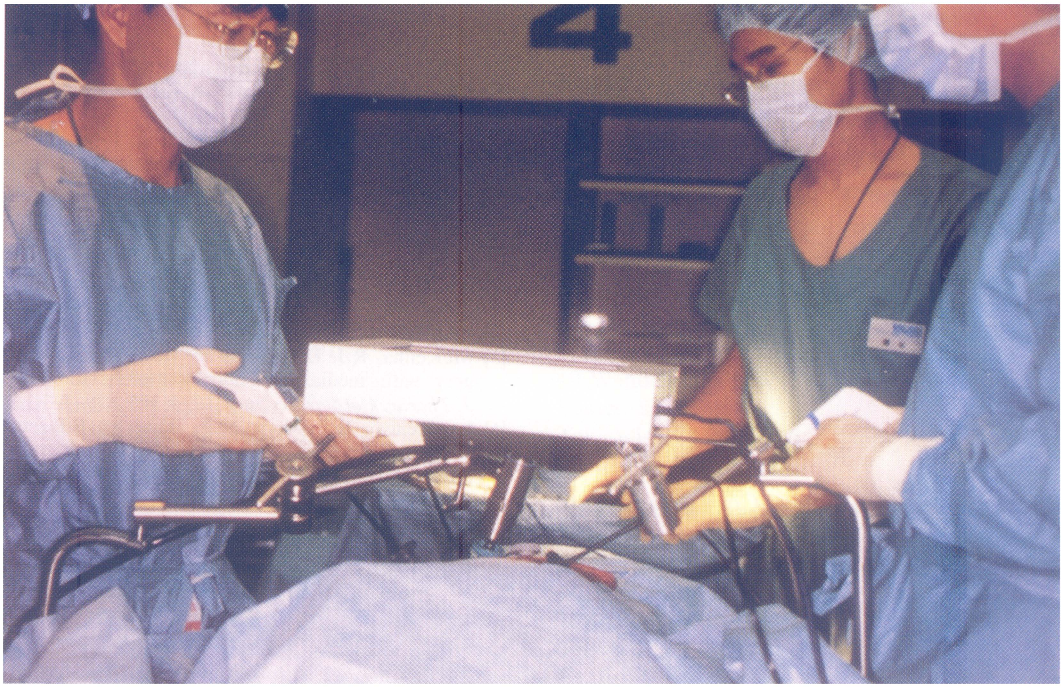

FIGURE 2 New setup; the monitor is placed above the patient.

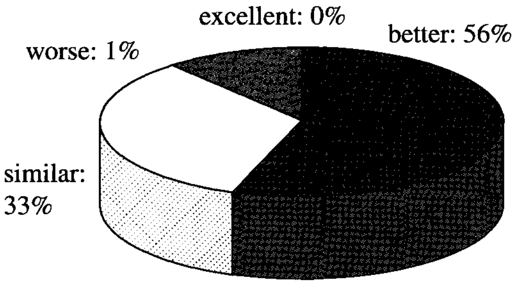

FIGURE 3 Judgment of the new setup system compared with the conventional one.

that the new setup was better than the conventional one (Fig. 3). One tester judged this system to be worse because of the difficulties in operating the original thoracoscope.

### DISCUSSION

In the conventional standard setup of VATS, two video monitors are necessary [6,7]. One monitor is placed opposite to the operator, near the patient's head, which allows the operator to view the monitor while looking straight ahead. Another video monitor is placed behind the operator which allows the assistant and/or camera holder a direct view of the procedure. What differs in our new concept for the setup is that one display-monitor and the original thoracoscope are also necessary. In using this procedure, the operator and assistants can see the patient and the monitor at the same time. According to this new idea, the previous problem in the area of hand–eye coordination and the three-dimensional understanding of this procedure can be improved compared to the image of the conventional thoracoscopy, because it is not necessary for the operator and assistants to look up at the monitors. When the thoracoscopy was placed in an adequate position to resect the target pathology, this new system led to good and easy handling of instruments, as it was with the standard thoracotomy, although the mirror image effect sometimes occurred if the instruments and scope were placed in opposing directions. However, several problems especially significant improvement in the quality of display and functions of the thoracoscopy must be solved in order for this system to be used for the clinical application as soon as possible.

Unfortunately, we were unable to demonstrate the usefulness of the new display system compared with the conventional setup in the preliminary experience. However, we would like to find ways to improve our new display system for practical use in the future.

### *Acknowledgments*

The authors thank Asahi Optical Co. Ltd. and Auto Suture Japan Co. Ltd. for their exellent technical support in the development of the system.

### *References*

[1] Wakabayashi, A. Expanded applications of diagnostic and therapeutic thoracoscopy. *J. Thorac. Cardiovasc. Surg.* 1991; **102**: 721–723.

[2] Roviaro, G.R., Rebuffat, C., Varoli, F. *et al.* Videoendoscopic pulmonary lobectomy for cancer. *Surg. Laparo. Endo.* 1992; **2**: 244–247.

[3] Lewis, R.I., Caccavale, R.J., Sisler, G.E. *et al.* One hundred consecutive patients undergoing video-assisted thoracic operations. *Ann. Thorac. Surg.* 1992; **54**: 421–426.

[4] Kirby, T.J., Mack, M.J., Landreneau, R.J. *et al.* Initial experience with video-assisted thoracoscopic lobectomy. *Ann. Thorac. Surg.* 1993; **56**: 1248–1253.

[5] McKenna, R.J. Lobectomy by video-assisted thoracic surgery with mediastinal node sampling for lung cancer. *J. Thorac. Cardiovasc. Surg.* 1994; **107**: 879–882.

[6] Landreneau, R.J., Mack, M.J., Hazelrigg, S.R. *et al.* Video-assisted thoracic surgery; Basic technical concepts and intercostal approach strategies. *Ann. Thorac. Surg.* 1992; **54**: 800–807.

[7] Krasna, M.J. and Mack, M.J. *Equipment and Instrumentation. Atlas of Thoracoscopic Surgery*, Quality Medical Publishing, St. Louis, 1994; pp. 19–33.

